# The incidence rate of unresponsive thin endometrium in frozen embryo transfer cycles: A case-series of therapy with granulocyte colony stimulating factor

**DOI:** 10.18502/ijrm.v17i12.5797

**Published:** 2019-12-30

**Authors:** Shokouhosadat Miralaei, Mahnaz Ashrafi, Arezoo Arabipoor, Zahra Zolfaghari, Saeideh Taghvaei

**Affiliations:** ^1^ Department of Endocrinology and Female Infertility, Reproductive Biomedicine Research Center, Royan Institute for Reproductive Biomedicine, ACECR, Tehran, Iran.; ^2^ Department of Obstetrics and Gynecology, Faculty of Medicine, Iran University of Medical Science, Tehran, Iran.; ^3^ Shahid Akbarabadi Clinical Research Development Unit (ShACRDU), Iran University of Medical Science (IUMS), Tehran, Iran.; ^4^ Department of Epidemiology and Reproductive Health, Reproductive Epidemiology Research Center, Royan Institute for Reproductive Biomedicine, ACECR, Tehran, Iran.

**Keywords:** Granulocyte colony-stimulating factor, Cryopreservation, Embryo transfer, Endometrial diseases.

## Abstract

**Background:**

Treatment-resistant thin endometrium (TTE) during in-vitro fertilization is a relatively uncommon and challenging problem.

**Objective:**

The primary aim of the study was to assess the TTE rate during frozen embryo transfer (FET) cycles and the secondary aim was to evaluate the effect of intrauterine instillation of granulocyte colony stimulating factor (G-CSF) in these cases.

**Materials and Methods:**

In this cross-sectional study, all of the women who underwent FET cycles with hormonal endometrial preparation in Royan Institute from June 2015 to March 2018 were evaluated and all of the cases with TTE diagnosis (endometrial thickness < 7 mm after using high doses of estradiol) were included. In the eligible cases, 300 μgr of G-CSF was infused intrauterine. If the endometrium had not reached at least a 7-mm, a second infusion was prescribed within 48 hr later.

**Results:**

During the study, 8,363 of FET cycles were evaluated and a total of 30 infertile patients (0.35%) with TTE diagnosis were detected. Finally, 20 eligible patients were included. The changes of endometrial thickness after G-CSF therapy were significant (p < 0.001); however, the endometrial thickness did not reach 7 mm in nine patients (45%) and the embryo transfer was canceled.

**Conclusion:**

It was found that the rate of TTE during the FET cycle is very low and intrauterine perfusion of G-CSF has a potential effect to increase the endometrial thickness in these patients; however, the rate of cancellation was still high and poor pregnancy outcomes were observed.

## 1. Introduction

Endometrial growth is an essential step in endometrial receptivity and embryo implantation in assisted reproductive technique cycles (1). A thin endometrium of < 7 mm is associated with a decreased odds ratio of achieving pregnancy whether with pathologic causes or idiopathic cases (1, 2). In addition, it is a challenging subject with unclear real prevalence (3). The incidence rate between less than 1-2% during in-vitro fertilization (IVF) cycles were reported in previous studies (2, 4). Different types of treatment including increased dose of estradiol, low-dose human chorionic gonadotropin, tamoxifen, pentoxifylline, vitamin E, l-arginine, low-dose aspirin, nitroglycerin patches, vaginal sildenafil, acupuncture and neuromuscular electric stimulation, intrauterine infusion of granulocyte colony stimulating factor (G-CSF), and recently stem cell therapy have been evaluated in previous studies (5). Garcia-Velasco and colleagues in a review article concluded that it is not easy to provide a pragmatic, evidence-based method to assist clinicians and patients who are confused with the available information regarding management approaches for a refractory endometrium (5).

“G-CSF is a glycoprotein and known as colony stimulating factor 3 produced in vascular endometrium, macrophages, and other immunocytes that operate not as a growth factor but also as a cytokine (5). G-CSF promotes endometrial stromal cell decidualization via cyclic adenosine monophosphate mediator by apocrine and paracrine action and induces proliferation and differentiation of the human endometrium” (5). In medicine recombinant, G-CSF was used in immunology for various indications for a long time (6). It is a pro-angiogenic mediator and increases type 2 T helper cell cytokine secretion, recruitment of dendritic and T regulatory cells (6, 7). According to previous clinical studies, G-CSF can be considered as a factor preventing repeated miscarriages and implantation failures (8-10), and it has no impact on the chromosomal constitution of human embryos (11). From the first case report study by Gleicher and co-workers in 2011 (4), until now, five studies in IVF (12-16) and four in FET cycles (3, 6, 17, 18) have evaluated the effect of intrauterine infusion of G-CSF on thin endometrium; since the reported results have been controversial.

The present study was conducted at the Royan Institute to assess the treatment-resistant thin endometrium rate during FET cycles with hormonal endometrial preparation (as primary aim) and to evaluate the effect of intrauterine instillation of G-CSF in these cases (as secondary aim).

## 2. Materials and Methods

In this cross-sectional study, we evaluated all of the women who underwent frozen embryo transfer (FET) cycles with hormonal *endometrial *preparation at the Royan Institute from June 2015 to May 2018, and all eligible cases with treatment-resistant thin endometrium diagnosis were included. The women aged 20-40 years with previously canceled at least one cycle because of thin unresponsive endometrium ( < 7 mm) during IVF or FET programs who had written consent to participate were included in the study. Other uterine abnormalities (such as Asherman's syndrome, fibroids, polyps, and adenomyosis diagnosis), cases with contraindications for G-CSF treatment (sickle cell disease, chronic renal and respiratory diseases...) and treatment cycles with embryo donation or prenatal genetic screening were excluded.

All women received three estradiol valerate tablet (2 mg, Aburaihan Co., Tehran, Iran) daily and low-dose aspirin from the second or third day of the menstrual cycle. The Endometrial thickness measurement (at its thickest part in the longitudinal axis of uterine) was carried out by ultrasonography, 9 or 10 days later. When the endometrial thickness was < 7 mm after using high doses of estradiol (8 mg), the same ultrasonographers measured it repeatedly for three times to approve thin endometrium, and the average size of the three different measurements was accepted. When the thin endometrium was diagnosed, the G-CSF infusion was performed according to methods previously described by Gleicher * et al*. in their study (12). In summary, the content of the G-CSF ampule (300 mcg/1ml) (Neupogen^TM^, Filgastrim, Amgen Inc., Thousand Oaks, Canada) was aspirated into a 1-ml insulin syringe, and it was inserted to the endometrial cavity using a soft catheter (Labotec, Gottingen Germany); then the G-CSF was gently infused, while the catheter was slowly ambulated back and forth. Two days later, the thickness of endometrium was measured and if it was at least 7 mm, the patient received 100 mg intramuscular progesterone in oil (50 mg; Aburaihan Co., Tehran, Iran) for three days. According to women's age and the quality of embryos, two or three cleavage stage embryos were transferred. If not, a second, identical infusion of G-CSF was done and then two or three days later, the endometrial thickness was evaluated again, and if it was less than 7 mm, the embryo transfer was canceled. All endometrial thickness measurements and intrauterine infusions have been performed by a specialist.

### Ethical consideration

The research protocol was approved by the Institutional Review Board and the Ethics Committee of the Royan Institute (Ethics code/94/178). Each participating women signed informed consent.

### Statistical analysis

Data was statistically analyzed by using Statistical Package for the Social Sciences (SPSS Inc., Chicago, Illinois, USA) software version 20. Categorical data were expressed as number and percentage and numerical data as mean and standard deviation. The statistical analyses for parametric data were performed with the use of a paired *t*-test to compare means endometrial thickness in patients before and after treatment according to the results of the Kolmogorov-Smirnov normality test for endometrial thickness. The level of statistical significance was set at p < 0.05.

## 3. Results 

During the study period, 8,363 FET cycles were evaluated and a total of 30 infertile patients (0.35%) with treatment-resistant thin endometrium diagnosis were detected, finally, 20 eligible patients were satisfied to receive the G-CSF therapy. Two patients did not agree to participate and eight patients did not meet the inclusion criteria of the study. The basic and demographic characteristics of patients are presented in Table I, Table II shows the changes in endometrial thickness after treatment with G-CSF. The mean of endometrial thickness at the time of infusion was 5.35 ± 1.06 mm, after intrauterine instillation of G-CSF, the mean of endometrium thickness was 6.52 ± 1.10 and the changes were significant (p < 0.001). Despite the increase in endometrial thickness after G-CSF injection, the endometrial thickness did not reach 7 mm or more in nine patients (45%) and the embryo transfer was canceled. Therefore, 11 patients had embryo transfer and no case of positive pregnancy was reported.

**Table 1:** Demographic characteristics of study participate (n = 20)


**Variables**	**Outcome**
Age (yr)*	35.2 ± 4.7
BMI (Kg/m^2)^*	26.2 ± 4.2
Basal serum level of LH*	5.3 ± 3.5
Basal serum level of FSH (mIU/ml)*	5.9 ± 1.4
Serum level of Prolactin (mIU/L)*	169.2 ± 147.7
Basal serum level of Estradiol (pg/ml)*	50.2 ± 43.3
Serum level of TSH (uU/ml)*	1.8 ± 0.7
Serum level of AMH (ng/ml)*	5.8 ± 4.0
Cause of infertility	
Ovulatory**	7 (35%)
Uterine factor**	1 (5%)
Male factor**	9 (45%)
Unexplained**	4 (20%)
Tuboperitoneal**	2 (5%)
Failed prior IVF cycles (n)*	1.4 ± 0.85
Duration of infertility (yr)*	8.1 ± 6.5
Prior history of a thin endometrium*	1.1 ± 0.36
*Data presented as Mean ± SD; **data presented as n (%)	
Descriptive statistics are used to describe the basic features of the data
BMI: Body mass index; LH: Luteinizing hormone; FSH: Follicle stimulating hormone; TSH: Thyroid stimulating hormone; AMH: Anti-Müllerian hormone; IVF: In-vitro fertilization

**Table 2:** Endometrial thickness in patients before and after treatment with G-CSF (n = 20)


**Variables**	**Outcome**	**P-value**
Endometrial thickness at the day of G-CSF infusion	5.35 ± 1.06	
Endometrial thickness at the day of embryo transfer	6.52 ± 1.10	
Δ Endometrial thickness	1.173 ± 1.10	< 0.001*
Data presented as Mean ± SD, Paired sample t-test was used for analysis, G-CSF: Granulocyte colony stimulating factor		

## 4. Discussion

In the present study, the incidence rate of thin endometrium during FET cycle in our institute was estimated to be 0.35%, which is almost similar to the rate of thin endometrium in IVF cycles (< 1%) reported by Al-Ghamdi and colleagues (19). It has been found that intrauterine injection of G-CSF in patients with unresponsive thin endometrium diagnosis can improve the endometrial thickness, but it failed to find out its effect on pregnancy. However, multiple factors affect pregnancy and endometrial receptivity and endometrial thickness are only a quantitative criterion; poor pregnancy outcomes in the present study can be due to that the averages of women's age and BMI were higher than those in other studies.

After the first case report study by Gleicher and co-workers (4), four case-series studies have evaluated the effect of intrauterine infusion of 300 μg of G-CSF in patients with thin endometrium diagnosis at the day of ovum pickup and reported a significant positive impact on improving endometrial thickness (12, 14-16). However, Check *et al*. in a small number of patients with unresponsive thin endometrium did not find significant effect after intrauterine infusion of G-CSF (20). Recently, Barad and colleagues in a randomized clinical trial assessed 73 normal patients during IVF and concluded that in normal IVF patients, G-CSF has no impact on the endometrial thickness, implantation rates, and clinical pregnancy rates (13).

A few studies evaluated the efficacy of intrauterine instillation of G-CSF for improving endometrial thickness in FET cycles, and yet the findings remain controversial. At first, Li *et al*. in a prospective study found no significant differences in the endometrial thickness, implantation, and clinical pregnancy rates between patients treated with 100 μg of G-CSF and the control subjects (3). Besides, Eftekhar and co-workers in a non-randomized clinical study assessed 68 patients with thin endometrium and reported no significant improvement in endometrial thickness; it is worth noting that the clinical pregnancy rate was higher in the G-CSF group than that in the control group (18). Later, Xu and colleagues in a prospective study divided 30 patients with thin endometrium diagnosis in two treatment subgroup (16 patients with G-CSF and 14 patients with G-CSF plus endometrial scratch) (17). After treatment in both the groups, the endometrial thickness increased significantly. The G-CSF with endometrial scratch subgroup associated with nominally higher rates of clinical pregnancy and live birth in comparison with the G-CSF only subgroup; though the differences were non-significant (17). Finally, it concluded that endometrial scratch did not damage the G-CSF action for thin endometrium and lean toward a better pregnancy and live birth rates. They recommended embryo transfer cancellation and G-CSF therapy in subsequent FET cycles for patients with a thin endometrium (17). In contrast, Kunicki and co-workers (6) evaluated 62 women with thin unresponsive endometrium (29 patients treated with a G-CSF infusion and 33 cases who opted out of the study considered as controls). It was found that G-CSF infusion leads to an improvement in endometrium thickness but has no beneficial effect on clinical pregnancy and live birth rates. Therefore, although the case series and non-randomized studies are promising, the only published randomized clinical trial failed to find any effect on clinical outcome. Reliable evidence for the beneficial effect of G-CSF in improving endometrial receptivity is still limited (5) and more data is warranted for a definitive conclusion in this regard.

The first limitation of the study is a relatively small cohort size; although, in most studies conducted in this area (3, 12, 14-16, 18), the patients' number in the experimental group (G-CSF treatment) was less than 40, due to the low prevalence of treatment-resistant thin endometrium (0.1-2%). Therefore, the evaluation of this number of patients can be valuable and represent a "small piece" to increase the research about this type of treatment. The second limitation is the lack of a control group. The strength of this study was to evaluate the incidence rate of thin endometrium during FET cycles since there is very limited data in this regard.

## 5. Conclusion 

It was found that the rate of treatment-resistant thin endometrium during the FET cycle with hormonal endometrial preparation is very low and G-CSF has a potential effect to increase the endometrial thickness in these patients; however, the rate of cancellation was still high and poor pregnancy outcomes were observed.

##  Conflicts of Interest 

There are no conflicts of interest to declare.
